# Stress Adaptation Phenomena of *Rhododendron* Species in Alpine Tundra and Timberline of Changbai Mountain: Physiological Traits and Molecular Evolution

**DOI:** 10.3390/plants14223528

**Published:** 2025-11-19

**Authors:** Zhongzan Yang, Jian You, Jiangnan Li, Wei Zhao, Ming Xing, Yujiao Zhang, Cui Ma, Yuqiao Gong, Yueming Zhao, Alimu Wubuli, Xia Chen

**Affiliations:** National & Local United Engineering Laboratory for Chinese Herbal Medicine Breeding and Cultivation, School of Life Sciences, Jilin University, Qianjin Avenue 2699, Changchun 130012, China; yangzhongzan902@163.com (Z.Y.);

**Keywords:** *Rhododendron*, alpine tundra, positive selection, adaptation, Changbai Mountain

## Abstract

In the context of climate change, *Rhododendron* species are pivotal in sustaining the stability of alpine ecosystems. Within alpine tundra (elevation > 2200 m) and timberline (elevation ~ 2000 m) regions of Changbai Mountain, the three studied *Rhododendron* species (*Rhododendron aureum*, *Rhododendron lapponicum*, and *Rhododendron redowskianum*) are prevalent; their mechanisms of adaptation to high-altitude environments remain insufficiently understood. This study employed an integrative approach, combining soil chemical analysis, physiological assessments, and molecular evolutionary analysis, to investigate phenotypic plasticity and genetic adaptation of these *Rhododendron* species. Both habitats demonstrated oligotrophic characteristics, with no significant differences (*p* > 0.05) observed in the concentrations of soil total organic carbon (TOC), ammonium nitrogen (NH_4_^+^-N), nitrate nitrogen (NO_3_^−^-N), and available phosphorus (AP). Nonetheless, soil nutrient variability was more marked in timberline. Physiological traits, including malondialdehyde (MDA), soluble sugar, proline, and soluble protein, exhibited species-specific patterns; for example, *R. redowskianum* displayed elevated proline content in the timberline habitat, although no consistent inter-habitat trends were identified. From a total of 1995 orthogroups analysed, we identified 279 positively selected genes (PSGs, *dN*/*dS* > 1). These genes were found to be enriched in GO terms associated with DNA replication, amino acid transport, and pathway of nucleocytoplasmic transport. The study highlights tissue development and reproduction as primary evolutionary trajectories, while identifying cold stress as a significant environmental selection pressure. This research elucidates *Rhododendron*’s alpine adaptability and provides insights into alpine plant adaptation mechanisms and species conservation under climate change.

## 1. Introduction

Species of the genus *Rhododendron*, renowned for their ecological plasticity, often function as pioneer species in successionally dynamic ecosystems. They demonstrate a remarkable capacity to adapt to a wide range of harsh environmental conditions, which is evidenced by their circumboreal distribution and presence across all continents except Antarctica [1]. In damaged forest ecosystems, *Rhododendron* species can rapidly take over bare soil, forming dense thickets. These thickets effectively mitigate soil erosion, enhance water retention, and facilitate the subsequent establishment of other plant species by modifying microclimatic conditions and improving edaphic properties [2]. In addition, species of the genus *Rhododendron* show robust survival at high altitudes and are able to thrive in extreme environments, including low temperatures, high ultraviolet (UV) radiation, strong winds, and nutrient-poor substrates [3]. This adaptation allows *Rhododendron* to play an important role in high-altitude ecosystems, especially in the context of climate change and increasing environmental pressures.

Studies have shown that the ability of *Rhododendrons* to survive and reproduce is closely related to their adaptation to extreme climatic conditions [4]. For instance, in biodiversity hotspots like the Tibetan Plateau and the Hengduan Mountains, the distribution of *Rhododendrons* was intricately linked to geological and climatic changes [5]. Furthermore, *Rhododendron* species demonstrated expanded gene families related to pathogen defense and oxidative phosphorylation, indicative of genomic adaptation and evolution in high-altitude environments [3]. However, it is not known whether *Rhododendron* species show adaptive evolution in other directions in extreme alpine environments. As one of the few regions in East Asia that preserves intact alpine tundra and timberline ecosystems, Changbai Mountain has *Rhododendron* communities (e.g., *Rhododendron aureum* (*R. aureum*)), *Rhododendron lapponicum* (*R. lapponicum*) and *Rhododendron redowskianum* (*R. redowskianum*). These communities serve as typical representatives of high-altitude adaptive populations [6]. This mountain provides an ideal natural research platform for investigating plant stress adaptation [7,8]. Its alpine tundra (elevation > 2200 m) and timberline (elevation ~ 2000 m) are characterized by unique environmental stressors. These stressors include: (1) low annual mean temperature (−7.3 to 2.2 °C), with frequent frost events even in summer [9,10]; (2) intense ultraviolet-B (UV-B) radiation [11,12]; (3) slow soil nutrient cycling, with organic matter decomposition rates only 1/3–1/2 of those in low-altitude temperate forests, leading to persistent nutrient limitation [13,14]. These extreme conditions make Changbai Mountain an ideal natural platform to study the adaptive mechanisms of high-altitude plants. Notably, *Rhododendron* species (e.g., *R. aureum*, *R. lapponicum*) are the dominant dwarf shrubs in Changbai Mountain’s alpine tundra and timberline [6,15]. Their ability to persist in these habitats makes them a key model to unravel high-altitude adaptation, yet the integrated mechanisms linking their growth, physiology, and molecular evolution remain unclear. Positively selected genes (PSGs) in plants constitute the molecular foundation for physiological adaptation, with the latter representing the phenotypic manifestation of the functional expression of PSGs [16]. These elements are interconnected through a causal sequence: environmental stress leads to PSG selection, which in turn results in functional specialization, ultimately culminating in the optimization of physiological phenotypes [17].

In alpine meadows and subalpine ecosystems, the presence of *Rhododendrons* not only enriched the diversity of plant communities, but also provided habitat for other species [18]. Therefore, the conservation of these alpine ecosystems and their pioneer plants, such as *Rhododendrons*, is crucial for maintaining biodiversity and ecological balance [19]. With global warming, Changbai Mountain’s alpine tundra has experienced a 1.14 °C increase in annual mean temperature over the past 30 years, leading to upward shifts of the timberline [10,20]. To the best of our knowledge, which PSGs in the *Rhododendron* genus respond to temperature stimuli and even severe ecological stress remains unknown. Previous studies have identified related PSGs from aspects such as flower color and anthocyanin synthesis [3,21]. This habitat change may disrupt *Rhododendron*’s adaptive strategies, making it urgent to clarify their current adaptation mechanisms to predict their future distribution. In this regard, we have explored the growth state and adaptive evolutionary direction of *Rhododendron* species in the context of alpine tundra and timberline. These explorations aim to provide new insights into the adaptation of *Rhododendron* species under climate change. Specifically, we have addressed three key scientific questions: (1) What are the differences in soil total organic carbon (TOC) and selected soil available nutrients between alpine tundra and timberline? (2) Do *Rhododendron* species exhibit species-specific physiological adaptation strategies in response to alpine tundra? (3) Which molecular pathways (via PSGs) underpin the phenotypic and physiological adaptation of *Rhododendron* to extreme alpine environments?

## 2. Results

### 2.1. Sampling Sites and Soil Chemical Properties

**Figure 1:**
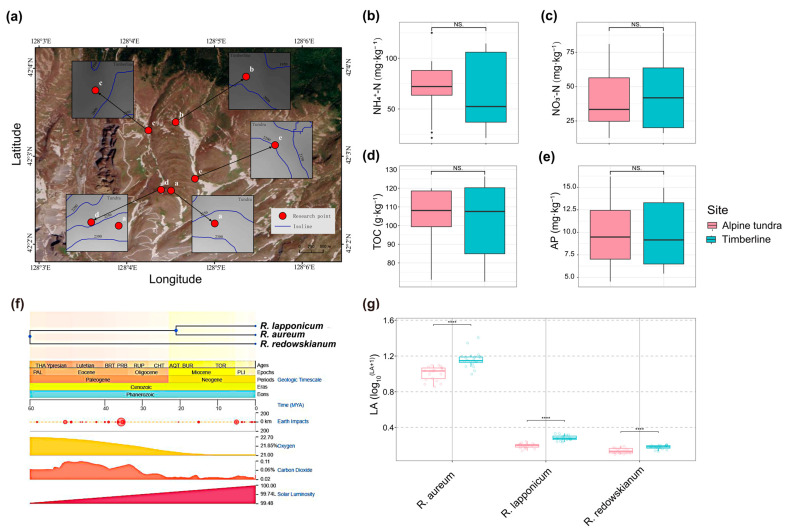
Information on sampling sites and plant samples. (**a**) Sampling sites in alpine tundra and timberline of Changbai Mountain. Red dots represent research plots and field sampling locations. Contents of soil ammonium nitrogen (NH_4_*^+^*−N) (**b**), nitrate nitrogen (NO_3_*^−^*−N) (**c**), total organic carbon (TOC) (**d**), and available phosphorus (AP) (**e**) in the sampling sites. For soil samples, n = 14. The Wilcoxon rank-sum test was used to evaluate the significance of differences (**** *p* < 0.0001 (extremely highly significant); NS, Not Significant, *p* ≥ 0.05) and the tops and bottoms of the boxes show the 75th and 25th percentiles, respectively. The same applies to the annotations below. (**f**) Phylogenetic tree illustrating the evolutionary relationships among the three studied *Rhododendron* species: *R. aureum*, *R. lapponicum*, and *R. redowskianum* (https://timetree.org/, accessed on 25 October 2025). (**g**) Boxplots of leaf area (denoted as LA) across *R. aureum*, *R. lapponicum* and *R. redowskianum* (n = 20).

The distribution of sampling sites and soil chemical properties (NH_4_^+^-N, NO_3_^−^-N, TOC, and AP) in alpine tundra and timberline habitats of Changbai Mountain was characterized (Figure 1a). Analysis of key soil nutrients revealed a generally oligotrophic condition in both habitats. NH_4_^+^-N (Figure 1b), NO_3_^−^-N (Figure 1c), and AP concentrations (Figure 1e) are lower in both alpine tundra and timberline habitats, with no significant differences (*p* > 0.05) between the two habitats. However, timberline soils exhibit greater variability as indicated by larger standard deviations and interquartile ranges in ammonium nitrogen, nitrate nitrogen, and available phosphorus compared to alpine tundra soils. Additionally, soil TOC (Figure 1d) content does not differ significantly (*p* > 0.05) between alpine tundra and timberline, reflecting the shared constraint of slow decomposition rates under low temperatures. growing in alpine tundra and timberline habitats were collected (Figure 1f), and we observed significant differences (*p* < 0.05) in their leaf area between these two habitats. Leaf area of *Rhododendron* species is larger in the timberline than in alpine tundra (Figure 1g, Table S1). This convergent morphological shift suggests a prominent environmental filtering effect on this particular trait. This, in turn, prompts us to investigate whether this is mirrored by consistent shifts in physiological and molecular adaptations. In light of this, we further investigated the physiological adaptation indices of these *Rhododendron* species.

### 2.2. Adaptive Phenomena of Rhododendron Species in Harsh Alpine Environments

#### 2.2.1. Leaf Physiological Adaptive Characteristics and Expression Levels of PSGs

**Figure 2:**
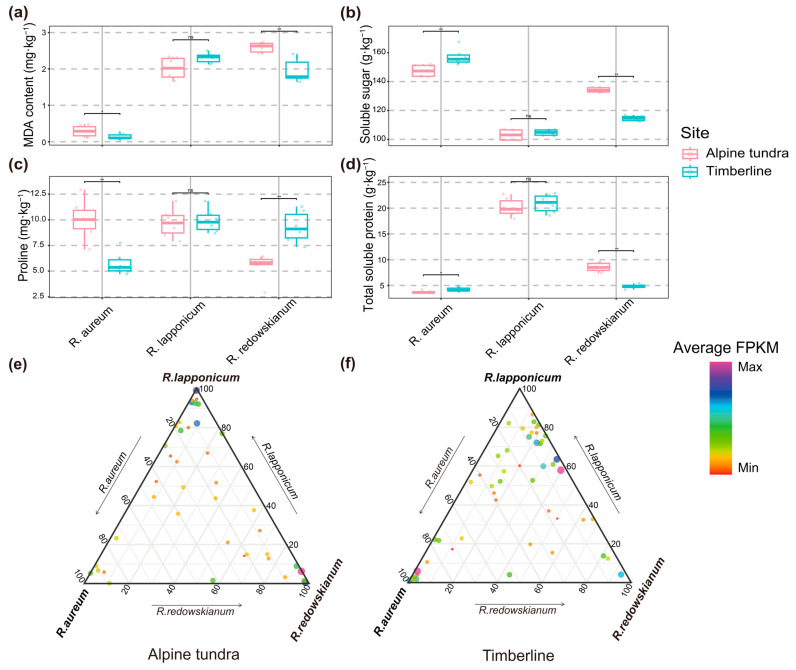
Adaptive characteristics and gene expression levels of *Rhododendron* plants in alpine tundra and timberline habitats. Leaf contents of MDA (malondialdehyde) (**a**), soluble sugar (**b**), proline (**c**), and total soluble protein (**d**) in plant leaves (n = 6). The Wilcoxon rank-sum test was used to evaluate the significance of differences (** *p* < 0.01 (highly significant); * *p* < 0.05 (significant); ns, Not Significant, *p* ≥ 0.05) and the tops and bottoms of the boxes show the 75th and 25th percentiles, respectively. Expression values of PSGs of the three studied *Rhododendron* species in alpine tundra (**e**) and timberline (**f**).

We analyzed leaf physiological indices—including MDA (Figure 2a), soluble sugar (Figure 2b), proline (Figure 2c), and total soluble protein (Figure 2d), as well as the expression levels of PSGs in *Rhododendron* species collected from alpine tundra (Figure 2e) and timberline (Figure 2f). When examining physiological adaptations, we found that *Rhododendron* species lack consistent inter-habitat differences. For instance, proline content in *R. redowskianum* and soluble sugar content in *R. aureum* were higher in timberline than in alpine tundra (Figure 2b,c); this variation underscores the diverse adaptive strategies employed by different *Rhododendron* species. This species-specific pattern underscores the diversity of physiological strategies employed by congeneric species to cope with environmental stress. Analysis of transcript expression levels for the identified PSGs confirmed that these genes were transcriptionally active in all three species in both tundra and timberline habitats (Figure 2e,f), indicating their functional relevance. Notably, plant leaf area were generally larger in timberline than in alpine tundra—a pattern that contrasts with the inconsistent trends observed for physiological indices [22]. Collectively, these findings indicate that distinct *Rhododendron* species utilize unique physiological adaptation mechanisms. These species-specific strategies ultimately drive clear differentiation in leaf area between alpine tundra and timberline habitats. To further unravel the molecular basis of *Rhododendron* species’ adaptation to harsh alpine conditions, we conducted selection pressure analysis (to identify PSGs) and subsequent characterization of PSGs.

#### 2.2.2. Functional Annotation of PSGs in *Rhododendron* Species

**Figure 3:**
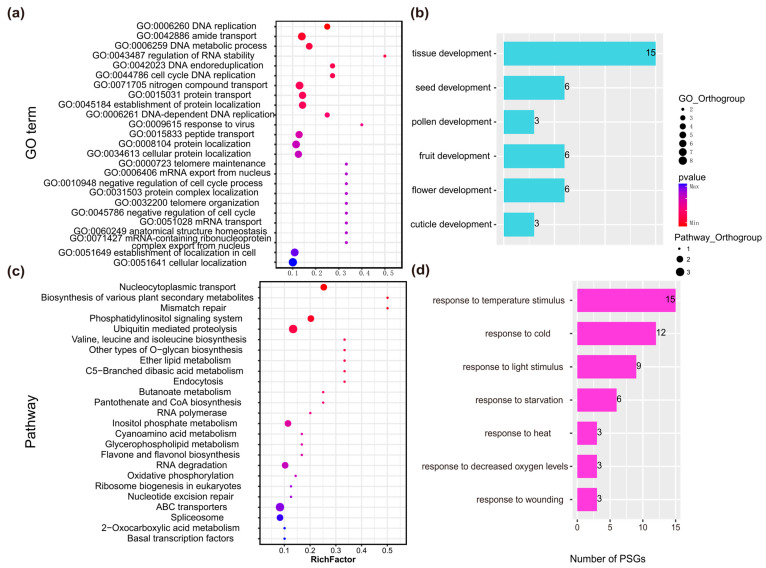
Annotation information related to PSGs. (**a**) Top 25 of GO term annotations of orthogroup. (**b**) Annotations of PSGs related to development. (**c**) Top 25 of KEGG annotations of orthogroup. (**d**) Annotations of PSGs related to stimulus.

To gain insights into the adaptability of *Rhododendron* species to harsh alpine environments, we performed selection pressure analyses on these *Rhododendron* species sampled from timberline and tundra habitats. A total of 279 PSGs were identified based on the criterion of *dN*/*dS* > 1 (Table S2). The GO annotation results show that functional modules such as DNA replication, amino acid transport, metabolic processes, and RNA stability regulation were significantly enriched. This suggests that these biological functions are the core functional targets for *Rhododendron* species to adapt to tundra and timberline, and their genes have undergone intense positive selection during evolution (Figure 3a, Table S3). For the GO annotation of developmental process (Figure 3b, Table S4), the number of PSGs related to tissue development (GO:0009888) was the largest, followed by those related to seed (GO:0048316), fruit (GO:0010154), and flower development (GO:0009908), and the number of PSGs related to pollen (GO:0009555) and cuticle development (GO:0042335) was the smallest. This indicates that tissue development and reproduction-related developmental processes (seed, pollen, fruit, and flower development) may be the key evolutionary directions for *Rhododendron* species to adapt to heterogeneous habitats. Among the genes for tissue development that underwent the greatest degree of positive selection, the reproductive developmental processes (seed, flower, fruit, etc.) also optimized the reproductive program through positive gene selection.

KEGG annotation results (Figure 3c, Table S5) show that pathways such as nucleocytoplasmic transport, biosynthesis of various plant secondary metabolites, and phosphatidylinositol signaling system (ko04070) were significantly enriched. This indicates that processes like substance transport, signal transduction, and metabolic regulation involved in these pathways are key molecular pathways for *Rhododendron* species to adapt to habitats. We also examined the distribution of PSGs related to stimulus responses (Figure 3d, Table S6). The number of PSGs responsive to temperature stimuli (GO:0009266) was the largest, followed by those responsive to cold (GO:0009409). There were also PSGs responsive to light stimuli (GO:0009416) and starvation (GO:0042594), as well as those responsive to heat (GO:0009408), decreased oxygen (GO:0036293), and wounding (GO:0009611). This reflects the core role of PSGs in environmental stress adaptation, especially temperature-related stress. This indicates that *Rhododendron* species have enhanced their adaptability to habitat pressures such as low temperatures and resource fluctuations in tundra and timberline by regulating the positive selection of stress-responsive genes.

## 3. Discussion

Alpine tundra and timberline habitats represent extreme environments. They are characterized by low temperatures, intense radiation, and nutrient-poor soils, which pose substantial challenges to plant survival and adaptation. This study investigated the adaptive strategies of *Rhododendron* species in these habitats. It integrated soil chemistry, physiological traits, and molecular evolution (PSGs) to elucidate the multi-level mechanisms underlying their persistence. Our integrative study combines edaphic, physiological, and molecular evolutionary analyses. It provides a holistic perspective on the adaptive strategies employed by *Rhododendron* species to persist in challenging ecosystems.

### 3.1. Soil Nutrients and Physiological Adaptation of Rhododendron Species

Consistent with previous findings on alpine soils [23,24], our results showed similar and low soil nutrient contents between alpine tundra and timberline. These nutrients include ammonium nitrogen, nitrate nitrogen, available phosphorus, and organic carbon (Figure 1b–e). This similarity likely stems from their close geographical proximity. It also reflects shared alpine environmental constraints, such as slow decomposition and nutrient cycling under low temperatures. The lack of significant differences suggests that the two habitats share fundamental constraints. These constraints affect nutrient mineralization and cycling, which are imposed by low temperatures. However, the greater variability in inorganic N and P availability observed in timberline soils exhibited greater variability in ammonium nitrogen, nitrate nitrogen, and available phosphorus compared to alpine tundra soils. This may reflect the more heterogeneous plant cover and litter inputs in this ecotone, creating a wider range of microsite conditions.

Nevertheless, for physiological adaption, the physiological data tell a more nuanced story. The absence of consistent inter-habitat trends in MDA, soluble sugar, proline and total soluble protein across the three species (Figure 2a–d) indicates that there is no single, universal physiological “syndrome” for coping with the conditions of tundra versus timberline. *Rhododendron* species exhibited species-specific patterns in physiological traits between the two habitats. Specifically, certain traits showed divergent trends across species; for instance, proline content in *R. redowskianum* and soluble sugar content in *R. aureum* were even higher in timberline than in alpine tundra (Figure 2b,c). Instead, each species appears to utilize a distinct combination of physiological adjustments. For instance, the elevated proline in *R. redowskianum* and soluble sugars in *R. aureum* in the timberline suggest species-specific osmotic adjustment strategies. This pattern aligns with timberline’s position as an “ecotone” between forest and alpine tundra, where species are exposed to dual habitat influences and greater biotic heterogeneity [25]. This posits that transition zones like the timberline harbor greater biotic and abiotic heterogeneity, potentially selecting for a wider array of adaptive strategies. Carbohydrates serve as key metabolites for plants adapting to low-temperature conditions, as they can lower the freezing point, mitigate intracellular ice formation, and stabilize the structures of biomembranes and proteins [26]. Proline, a classic osmotic adjustment substance, is capable of preventing dehydration-induced damage and alleviating oxidative damage under cold stress [27]. Furthermore, it functions as a “molecular chaperone” that binds to biological macromolecules (e.g., enzymes and nucleic acids), thereby sustaining the basic metabolic activities of plants [28]. Moreover, interspecific differences in stress tolerance likely contributed to the divergent physiological responses [29].

### 3.2. The Molecular Mechanisms of Adaptation of Rhododendron Species

A total of 279 PSGs were identified in *Rhododendron* species inhabiting alpine tundra and timberline ecosystems, offering critical molecular insights into their long-term adaptive strategies in response to the rigorous conditions of high-altitude environments. In contrast to neutral genetic markers, PSGs provide a direct reflection of the evolutionary pressures imposed by specific ecological challenges. This characteristic of PSGs enables the correlation of genomic attributes with functional traits that enhance survival and reproductive success in extreme environments. The subsequent discussion will explore the biological significance of the functional annotation results. It will then analyze these findings through the integration of existing literature, and elaborate on their implications for understanding the adaptive mechanisms of alpine flora.

#### 3.2.1. Metabolism and Basic Cellular Processes Are the Core Foundation of Adaptation

PSGs exhibit significant enrichment in functional modules associated with DNA replication, amino acid transport, metabolic processes, and RNA stability regulation (Figure 3a). This enrichment underscores the critical role of sustaining core cellular functions under high-altitude stress conditions. High-altitude environments are typically characterized by limited nutrient availability, such as nitrogen deficiency in tundra soils (Figure 1b,c), and low temperatures [22], which can affect biochemical reactions [30,31]. These stressors pose direct challenges to cellular metabolism and genetic stability. For example, the function of “amino acid transport” may play a major regulatory role in the balance between growth and induced resistance [32,33]. Similarly, PSGs related to RNA stability are crucial for ensuring the proper translation of key stress-responsive genes [34]. The enrichment of PSGs associated with DNA replication indicates that plants have enhanced their capacity to maintain genomic integrity. This trait, while often underemphasized in alpine adaptation research, is essential for survival at high altitudes. Consequently, positive selection on genes involved in DNA replication may represent an evolutionary strategy to repair damage and ensure precise genome replication. This is particularly relevant during periods of active plant growth, such as the brief alpine summer. In conclusion, these findings suggest that *Rhododendron* species prioritize addressing the fundamental limiting factors of alpine environments by optimizing essential cellular processes.

#### 3.2.2. Tissue Plasticity and Reproductive Assurance Are Key Evolutionary Directions

Genes that have undergone positive selection and are associated with tissue development are the most prevalent (Figure 3b), followed by those linked to reproduction, including seed, fruit, and flower development. This pattern suggests that *Rhododendron* species have evolved an adaptation that prioritizes both “tissue development” and “reproductive success” in alpine environments, both of which are intricately connected to fitness. PSGs involved in tissue development play a crucial role in the formation of reproductive structures. PSGs related to reproduction, such as those governing seed and flower development, may be crucial for overcoming the distinctive challenges posed by alpine ecosystems. These challenges including short growing seasons and unpredictable pollinator availability. The PSGs may enhance reproductive efficiency primarily by optimizing reproductive timing, such as accelerating flower development to coincide with the brief warm period. Secondly, they may achieve this by improving seed viability—for instance, by thickening the seed coat to withstand freeze-thaw cycles.

#### 3.2.3. Temperature Is One of the Dominant Selective Pressures

PSGs are predominantly involved in pathways such as nucleocytoplasmic transport, plant secondary metabolite biosynthesis, and the phosphatidylinositol signaling system (Figure 3c). This underscores the importance of coordinating molecular signal transduction and specialized metabolite synthesis in alpine adaptation [35]. Nucleocytoplasmic transport facilitates the movement of proteins and RNA between the nucleus and cytoplasm. This process acts as a crucial mechanism for the rapid regulation of gene expression in response to environmental change [36]. In high-altitude environments, where temperatures can vary significantly within short periods, efficient nucleocytoplasmic transport allows *Rhododendron* species to swiftly activate stress-responsive genes. These genes include cold-responsive transcription factors. This process thereby prevents delays in nucleocytoplasmic signal transmission. The variations in soluble sugar and proline content illustrate the osmotic adjustment and stress-resilient solute accumulation strategies employed by *Rhododendron* in extreme environments, such as low temperatures. GO annotations reveal enrichment in functions like “nitrogen compound transport (GO:0071705)” and “protein localization (GO:0015031)”. These functions underpin the molecular mechanisms for the synthesis, transport, and subcellular localization of solutes, including proline and soluble sugars. Furthermore, KEGG annotations indicate enrichment in pathways related to the “biosynthesis of various plant secondary metabolites”. These secondary metabolites, such as flavonoids, exhibit antioxidant properties that facilitate the scavenging of reactive oxygen species and mitigate membrane lipid peroxidation, thereby reducing the production of MDA.

The enrichment of PSGs associated with temperature responses (particularly to cold) alongside genes responsive to light, starvation, hypoxia, and wounding is shown in Figure 3d. This enrichment corroborates the notion that temperature serves as a primary selective pressure in tundra and timberline ecosystems. PSGs responsive to cold may encompass key components of established cold signaling pathways. Conversely, those responsive to light are specifically adapted to address the stress of high-altitude UV radiation, which can damage photosynthetic machinery and DNA [37]. Additionally, PSGs associated with responses to starvation and hypoxia reflect other environmental constraints in alpine regions. These constraints include nutrient scarcity (starvation) due to slow soil decomposition and oxygen limitation (hypoxia) in waterlogged tundra soils. These environmentally stress-responsive PSGs are highly consistent with our physiological findings. Specifically, the selection pressure imposed by the harsh alpine environment promotes plants’ adaptation to the alpine habitat by regulating their antioxidant systems and the accumulation of osmolytes. In conclusion, the widespread presence of stress-responsive positively selected genes suggests that *Rhododendron* species have developed multiple adaptive strategies. This strategy not only targets the most severe stressor, low temperature, but also addresses secondary limiting factors, thereby enhancing fitness in the complex conditions of alpine environments. The Changbai Mountain tundra is small in area and lacks obvious geographic isolation. The key adaptive traits of tundra species (such as cold and response to temperature stimuli) are highly correlated and may be genetically regulated by the same set of PSGs (Table S6). However, global climate warming is driving rising temperatures and greater temperature fluctuations in alpine regions. These environmental shifts may trigger the breakdown of the existing regulatory mechanisms governing “cold-responsive PSGs” and thus expose *Rhododendron* species to new risks.

All leaf samples were collected on 2 August 2022, which may introduce climatic stochasticity. Previous relevant studies have suggested that the climatic conditions do not differ significantly from those at the end of July [22]. The complex interactions between these genes and their collective regulation of adaptive phenotypes remain poorly understood. Future work should focus on functional validation of key PSGs through transgenic approaches and monitoring the temporal dynamics of these adaptations in the face of rapid climate change. Undoubtedly, in the harsh environmental conditions of alpine tundra, plant symbiosis and soil rhizosphere microorganisms play a pivotal role in the adaptation process of *Rhododendron* species. Incorporating these microorganisms and symbiosis into future studies will further facilitate a more comprehensive understanding of the adaptation mechanisms of *Rhododendron*. This study has certain limitations, including the reliance on single-time-point sampling and the omission of rhizosphere microbial interactions. Future research should aim to address these limitations.

## 4. Materials & Methods

### 4.1. Experimental Materials and Sampling

Sample collection was conducted in two key habitats of Changbai Mountain, a national nature reserve that boasts excellent natural landscapes [38]. These habitats were selected based on the principle of topographic similarity. The two habitats are (1) alpine tundra (42.043–42.045° N, 128.075–128.081° E) at an elevation of 2250 ± 25 m; and (2) timberline (42.054–42.056° N, 128.071–128.074° E) at an elevation of 2000 ± 20 m (see Figure 1a for geographic locations). At each sampling site in alpine tundra and timberline, quadrats were set according to plant types. For *Rhododendron* species, 2 m × 2 m square quadrats were established. More than 6 quadrats were established per *Rhododendron* species in tundra and timberline habitats, incorporating symbiosis into the observations. The distance between adjacent quadrats exceeded 5 m, which effectively avoided spatial autocorrelation and ensured the spatial representativeness of the samples. All composite samples were derived from independent biological replicates of different quadrats. Within each quadrat, detailed surveys and leaf collection were conducted for the three target species. During the survey, the following parameters were recorded for each *Rhododendron* individual in the quadrat: for leaf collection, mature, non-senescent leaves were selected from the middle-upper part of the plant (to avoid within-plant heterogeneity in leaf traits). More than 15 leaves were collected from each species per quadrat, and leaves from the same species in the same plot were mixed into one composite sample (total n = 6 composite samples per species per habitat). All leaf samples were immediately placed in liquid nitrogen after collection to inhibit physiological metabolism, and then stored at −80 °C in the laboratory until further analysis.

For plant samples, more than 15 mature, non-senescent leaves were collected from each of *Rhododendron* species: *R. aureum*, *R. lapponicum*, and *R. redowskianum*. Specific environmental conditions and sampling methods were followed as described in Yang et al., 2025 [22]. Mature, non-senescent leaves of *Rhododendron* species in timberline and alpine tundra were collected on 2 August 2022. This sampling time was chosen because early August is the peak growing season of alpine plants in Changbai Mountain, when leaves are fully developed and physiological activities are most active, ensuring that the measured traits can reflect the plant’s adaptive status. Soil samples were collected at a 10 cm depth from the soil surface adjacent to *Rhododendron* species to analyze chemical properties. For soil sampling, a 5-point sampling method was used within each quadrat: soil cores (diameter 5 cm, depth 10 cm) were collected at the four corners and center of the quadrat, mixed into one composite sample (n = 14 composite soil samples per habitat). After removing visible debris (e.g., roots, stones), each soil sample was air-dried at room temperature (25 °C) for 7 days, ground, and passed through a 2 mm sieve. These processed samples were then used to determine total organic carbon (TOC), ammonium nitrogen (NH_4_^+^-N), nitrate nitrogen (NO_3_^−^-N), and available phosphorus (AP).

### 4.2. Determination of Physicochemical and Physiological Indicators

#### 4.2.1. Soil Physicochemical Properties

Soil physicochemical properties were determined following protocols described in Soil Sampling and Methods of Analysis [39]. Specifically: TOC was measured using the K_2_Cr_2_O_7_ redox titration method. NH_4_^+^-N was determined via the indophenol blue colorimetric method. NO_3_^−^-N was analyzed using the phenol disulfonic acid colorimetric method. AP was extracted and quantified using the Olsen method.

#### 4.2.2. Leaf Area and Physiological Indicators

Leaf area (LA) was quantitatively measured using a Li-3000 leaf area meter (Li-Cor Biosciences, Lincoln, NE, USA), with more than 15 biological replicates. For better representation, the LA was subjected to logarithmic transformation. Malondialdehyde (MDA) content, total soluble sugars, proline content, and total soluble proteins were all determined on mature and non-senescent plant leaves. MDA content was measured as thiobarbituric acid-reactive material from leaf extracts (10% trichloroacetic acid) following centrifugation [40]. Total soluble sugars were extracted with 80% ethanol and determined using a modified protocol [41]. Proline content was quantified by the colorimetric method [42]. Total soluble proteins were measured using Coomassie brilliant blue staining [43]. Each physiological indicator was subjected to 6 biological replicates.

### 4.3. Identification and Annotation of Orthologs

#### 4.3.1. Identification of Single-Copy Orthologs

Previously published de novo transcriptome sequencing data (PRJNA1121965) were used to identify single-copy orthologous genes among *R. aureum*, *R. lapponicum*, and *R. redowskianum* (misidentified as *R. bracteatum*) using OrthoFinder (v2.5.4) [22,44]. OrthoFinder was used with default parameters, including: (i) sequence similarity search via DIAMOND (E-value ≤ 1 × 10^−5^) [45]; (ii) orthogroup clustering using the MCL algorithm with an inflation parameter of 1.5 [46]; and (iii) species tree calibration based on single-copy orthologs. Only orthogroups containing exactly one gene per species (i.e., single-copy in all three species) were retained, as these are critical for reducing bias in selection pressure analysis. A total of 1995 orthogroups were obtained after clustering, and all orthogroups were included in the subsequent selection pressure analysis, i.e., the total number of orthogroups analyzed in this study was 1995.

#### 4.3.2. Coding Sequence (CDS) Prediction

Sequences of each single-copy ortholog were extracted, and their CDS regions were predicted using a combination of BLASTx (v2.16.0) and ESTScan (v3.0.3-6) software [47,48,49]. To ensure accurate prediction of the coding sequence (CDS) regions, a dual-strategy was adopted. Sequences were first subjected to BLASTx searches against the NCBI non-redundant (nr) protein database to identify homologous proteins with known coding regions with an E-value threshold of 1 × 10^−5^. Subsequently, ab initio prediction was performed using ESTScan software to identify potential CDS in sequences lacking significant BLAST hits (E-value < 1 × 10^−5^). The predicted CDS were manually inspected for the presence of start and stop codons and the absence of internal stop codons.

#### 4.3.3. Multiple Sequence Alignment and Optimization

Predicted CDS sequences were aligned using MAFFT (v7.526) [50], which automatically selects the optimal alignment strategy (e.g., FFT-NS-1, FFT-NS-2, or L-INS-i) based on sequence size and divergence to balance accuracy and computational efficiency. Alignment results were refined and denoised with trimAL (v1.4.1) using the -automated1 parameter [51]. This parameter automatically identifies the appropriate method for trimming aligned sites based on sequence characteristics. It employs an automated heuristic method to remove poorly aligned regions and gaps. This process reduces noise and false positives in the detection of positive selection.

#### 4.3.4. Selection Pressure Analysis

The evolutionary relationships of the three species analyzed in this study, *R. aureum*, *R. lapponicum* and *R. redowskianum*, were obtained from Timetree (https://timetree.org/, accessed on 25 October 2025). After removing information such as divergence time, the data were used as the input file for the next step. Selection pressure on orthologs was assessed using PAML (v4.9) with the CodeML’s batch computing for the Branch-Site Model (BSM) [52]. A species tree was constructed using IQ-TREE from concatenated trimmed CDS alignments, with 1000 ultrafast bootstrap replicates to ensure topology reliability [53]. The alternative model (allowing positive selection in foreground branches: model = 2, NSsites = 2, fix_omega = 0, initial ω = 1.5) was compared to the null model (forbidding positive selection: fix_omega = 1, ω = 1). Significant results were identified via chi-square test (*p* < 0.05). The *dN*/*dS* ratio (nonsynonymous to synonymous substitution rate per site) was used to infer selection patterns: *dN*/*dS* < 1 (purifying selection), *dN*/*dS* ≈ 1 (neutral evolution), and *dN*/*dS* > 1 (positive selection) [54,55]. To account for multiple testing, Benjamini–Hochberg false discovery rate (FDR) correction was applied to raw *p*-values, and genes with FDR-corrected q-values < 0.05 were considered under significant positive selection [56].

#### 4.3.5. Functional Annotation

The expression profiles of PSGs were derived from the reanalysis of previously published data, with specific reference to Yang et al. [22]. Orthologous genes and their encoded proteins were annotated with Gene Ontology (GO) terms and Kyoto Encyclopedia of Genes and Genomes (KEGG) pathways using BLASTx with an E-value threshold of 1 × 10^−5^ [57,58]. The protein sequences encoded by the identified PSGs were functionally annotated by performing BLASTx searches against the Swiss-Prot and NCBI nr databases with a stringent E-value cutoff of 1 × 10^−5^. GO terms were assigned based on the best BLAST hits. For pathway analysis, the sequences were mapped to the KEGG database to identify enriched biological pathways.

### 4.4. Data Analysis

All statistical analyses were performed in R software (v4.3.1; http://www.r-project.org, accessed on 30 October 2025), including descriptive statistics and differential analysis of soil physicochemical indicators, leaf physiological parameters, and gene sequence data. The phylogenetic tree was constructed using the OrthoFinder rooted species tree [22,59]. Visualizations were generated using R packages: ternary plots with the ggtern package (v3.5.0; http://www.ggtern.com, accessed on 30 October 2025) and bar plots with the ggplot2 package (3.4.2) [60]. The Wilcoxon rank-sum test was used to evaluate the significance of differences [61]. As a nonparametric test, the Wilcoxon rank-sum test can more reliably determine the significance of intergroup differences. Data were visualized using specialized R packages: ternary plots for soil nutrient proportions were created with the ggtern package, and bar plots, boxplots, and other figures were generated using the ggplot2 package.

## 5. Conclusions

Our study examined the phenotypic plasticity and genetic adaptation of the three studied *Rhododendron* species to alpine tundra and timberline environments of Changbai Mountain. Physiological characteristics exhibited species-specific patterns. The 279 identified PSGs played a crucial role in molecular adaptation. These genes not only sustained essential cellular functions such as DNA replication and amino acid transport, but also optimized tissue and reproductive traits. In addition, they enhance stress signal transduction capabilities, such as nucleocytoplasmic transport, and specifically respond to low temperatures, which represent the predominant selective pressure. The study enhances our understanding of the ecological adaptability and adaptive trends of *Rhododendron*’s even alpine shrubs amid global climate change through selection pressure analysis.

## Data Availability

Data are available on request from the authors. Transcriptome sequencing data for *Rhododendron* species have been submitted to the Short Read Archive (SRA) data library under accession number: PRJNA1121965 (https://www.ncbi.nlm.nih.gov/sra/?term=PRJNA1121965) [22]. *R. bracteatum* refers to *R. redowskianum*, and the previous use of this name was a misapplication [22,62]. Code used for data analysis is available at https://github.com/18953239775/Denovo_RNA_PSGs.git.

## Supplementary Materials

The following supporting information can be downloaded at: https://www.mdpi.com/article/10.3390/plants14223528/s1, Table S1: Plant leaf area of thress *Rhododendron* species; Table S2: Positively selected genes in the genus *Rhododendron*; Table S3: GO term of orthogroup; Table S4: GO annotations for developmentally relevant PSGs; Table S5: KEGG pathway of orthogroup; Table S6: PSGs related to stimulus responses.

## Abbreviations

The following abbreviations are used in this manuscript:

PSGs	positively selected genes
GO	Gene Ontology
KEGG	Kyoto Encyclopedia of Genes and Genomes
SOC	soil total organic carbon
NH_4_^+^-N	ammonium nitrogen
NO_3_^−^-N	nitrate nitrogen
AP	available phosphorus
LA	leaf area
MDA	malondialdehyde
CDS	coding sequence
BSM	Branch Site Model
